# Supplementary material for: Empirical methods for controlling false positives and estimating confidence in ChIP-Seq peaks
Source: BMC Bioinformatics. 2008 Dec 5;9:523. doi: 10.1186/1471-2105-9-523 (PMC2628906; doi:10.1186/1471-2105-9-523)
Supplement: Additional File 1 — A variety of html documents from the USeq web site detailing the available applications, their best usage, output file type descriptions, command line menus, etc. [file 1471-2105-9-523-S1.zip › USeqUserGuides/JavaDoc.zip!/JavaDoc/trans/cel/class-use/Quantile.html]

Uses of Class trans.cel.Quantile


|  |  |  |
| --- | --- | --- |
| **Overview** \| **Package** \| **Class** \| **Use** \| **Tree** \| **Deprecated** \| **Index** \| **Help** | |  |
| PREV   NEXT | **FRAMES**    **NO FRAMES**     **All Classes** |  |


---


## **Uses of Class trans.cel.Quantile**

| Packages that use Quantile | |
| --- | --- |
| **trans.cel** | Apps and methods for manipulating cel files. |

Uses of Quantile in trans.cel

| Methods in trans.cel that return Quantile | |
| --- | --- |
| `Quantile[][]` | `QuantileNormalization.getQuantiles()`             Fetches an array of sorted normalized Quantiles |
| `static Quantile[][]` | `QuantileNormalization.makeQuantiles(float[][] values)`             Makes a Quantile[][] given float[][] where the quantile.position is assigned its array index position and the quantile.value is assigned the float value. |

| Methods in trans.cel with parameters of type Quantile | |
| --- | --- |
| `double` | `QuantileNormalization.calculateMedianOfChromosome(Quantile[] intensities, java.lang.String chromosomeName)`             Looks in the tpmapInfo for the appropriate chromosome, extracts the intensities from the Quantile[], calculates a median. |
| `float[]` | `QuantileNormalization.fetchControlIntensities(Quantile[] q)`             Uses the control indexes to fetch the control intensities from the Quantile[]. |
| `static double` | `QuantileNormalization.fetchMedianQuantileValue(Quantile[] m)`             Calculates the median intensity of a sorted Quantile[]. |
| `static void` | `QuantileNormalization.normalizeQuantileArrayScores(Quantile[][] quantiles)`             Takes arrays of Quantile, and averages the values for a given array index. |
| `static float` | `QuantileNormalization.percentile(Quantile[] sortedQs, double percentile)`             Returns the value of a given percentile from a SORTED Quantile array. |
| `static void` | `QuantileNormalization.scaleQuantileArraysByMedian(Quantile[][] quantiles, float targetMedian)`             Takes arrays of Quantile that have been sorted by value, and scales their values to the target median. |
| `void` | `QuantileNormalization.setQuantiles(Quantile[][] quantiles)` |
| `void` | `QuantileNormalization.sortQuantileArrays(Quantile[][] q)`             Sorts quantile arrays based on comparator, either position or intensity value. |

---


|  |  |  |
| --- | --- | --- |
| **Overview** \| **Package** \| **Class** \| **Use** \| **Tree** \| **Deprecated** \| **Index** \| **Help** | |  |
| PREV   NEXT | **FRAMES**    **NO FRAMES**     **All Classes** |  |


---
